# Bat flies (Diptera: Nycteribiidae and Streblidae) infesting cave-dwelling bats in Gabon: diversity, dynamics and potential role in *Polychromophilus melanipherus* transmission

**DOI:** 10.1186/s13071-016-1625-z

**Published:** 2016-06-10

**Authors:** Judicaël Obame-Nkoghe, Nil Rahola, Mathieu Bourgarel, Patrick Yangari, Franck Prugnolle, Gael Darren Maganga, Eric-Maurice Leroy, Didier Fontenille, Diego Ayala, Christophe Paupy

**Affiliations:** Laboratoire MIVEGEC, UMR 224–5290 CNRS-IRD-UM, IRD Montpellier, Montpellier, France; Centre International de Recherches Médicales de Franceville (CIRMF), Franceville, Gabon; Centre de coopération internationale en recherche agronomique pour le développement (CIRAD), UPR AGIRS, F-34398 Montpellier, France; Institut Pasteur du Cambodge, Phnom Penh, Cambodia

**Keywords:** Bat fly, Ectoparasites, Bats, mtDNA, Cytochrome *b*, *Polychromophilus*, Caves, Gabon, Central Africa

## Abstract

**Background:**

Evidence of haemosporidian infections in bats and bat flies has motivated a growing interest in characterizing their transmission cycles. In Gabon (Central Africa), many caves house massive colonies of bats that are known hosts of *Polychromophilus* Dionisi parasites, presumably transmitted by blood-sucking bat flies. However, the role of bat flies in bat malaria transmission remains under-documented.

**Methods:**

An entomological survey was carried out in four caves in Gabon to investigate bat fly diversity, infestation rates and host preferences and to determine their role in *Polychromophilus* parasite transmission. Bat flies were sampled for 2–4 consecutive nights each month from February to April 2011 (Faucon and Zadie caves) and from May 2012 to April 2013 (Kessipoughou and Djibilong caves). Bat flies isolated from the fur of each captured bat were morphologically identified and screened for infection by haemosporidian parasites using primers targeting the mitochondrial cytochrome b gene.

**Results:**

Among the 1,154 bats captured and identified as *Miniopterus inflatus* Thomas (*n* = 354)*, Hipposideros caffer* Sundevall complex (*n* = 285)*, Hipposideros gigas* Wagner (*n* = 317)*, Rousettus aegyptiacus* Geoffroy (*n* = 157*,* and *Coleura afra* Peters (*n* = 41), 439 (38.0 %) were infested by bat flies. The 1,063 bat flies recovered from bats belonged to five taxa: *Nycteribia schmidlii scotti* Falcoz*, Eucampsipoda africana* Theodor, *Penicillidia fulvida* Bigot, *Brachytarsina allaudi* Falcoz and *Raymondia huberi* Frauenfeld group*.* The mean infestation rate varied significantly according to the bat species (ANOVA, *F*_(4,75)_ = 13.15, *P* < 0.001) and a strong association effect between bat fly species and host bat species was observed. *Polychromophilus melanipherus* Dionisi was mainly detected in *N. s. scotti* and *P. fulvida* and less frequently in *E. africana, R. huberi* group and *B. allaudi* bat flies. These results suggest that *N. s. scotti* and *P. fulvida* could potentially be involved in *P. melanipherus* transmission among cave-dwelling bats. Sequence analysis revealed eight haplotypes of *P. melanipherus*.

**Conclusions:**

This work represents the first documented record of the cave-dwelling bat fly fauna in Gabon and significantly contributes to our understanding of bat fly host-feeding behavior and their respective roles in *Polychromophilus* transmission.

**Electronic supplementary material:**

The online version of this article (doi:10.1186/s13071-016-1625-z) contains supplementary material, which is available to authorized users.

## Background

Bats are among the most important reservoirs of pathogens that are major public health concerns worldwide [1–3]. The African continent hosts more than one third of all bat species (13 families, 70 genera and more than 340 species) [4]. Some species colonize cave environments, forming very large colonies that include up to several thousands of individuals, with a high degree of promiscuity that favors pathogen transmission. Besides viruses, bats are the hosts of several haemosporidian parasites [5–8], including parasites from the genera *Plasmodium* Marchiafava & Celli, 1885 [6, 9, 10], *Hepatocystis* Levaditi & Schoen, 1932 [11] and *Nycteria* Garnham & Heisch, 1953 [12], which mostly infect Yinpterochiroptera [13–15] and Yangochiroptera bats, including Miniopteridae. Earlier descriptions of haemosporidian parasites infecting cave-dwellings bats [13, 14, 16, 17] have raised a growing interest on their transmission cycles and their potential arthropod vectors. The vector role of several blood-sucking arthropod groups living in caves has been investigated, with particular emphasis on mosquitoes [5, 18–20], biting midges [21] and sand flies [22–25]. Conversely, only few studies have been devoted to bat flies (Diptera: Brachycera), despite their role in transmission of infectious agents [26] favored by their ectoparasitic lifestyle, and particularly their circumstantial incrimination in bat haemosporidian parasite infections [15, 27].

Bat flies infest exclusively bats and, like all members of the superfamily Hippoboscoidea, reproduce via viviparous puparity [28]. They belong to two families: Streblidae and Nycteribiidae*.* Streblidae bat flies have functional wings, while Nycteribiidae flies have a spider-like appearance and are wingless, as an extreme adaptation to ectoparasitism. Male and female bat flies are strictly haematophagous [29] and feed regularly on their hosts. Bat flies spend most of their lifetime on bats, nested in the fur and wings, and die within two days when they are separated from their host [30]. Females regularly leave their host to deposit prepupae, after which they colonize another host individual [29]. Generally, bat flies are monoxenous (i.e. they parasitize a single bat species) [31, 32]; however, they may infest more than one host species (oligoxenous, or polyxenous) [33, 34], depending on the host availability and behavior.

To date, nearly 40 species (or sub-species) of Nycteribiid and 32 of Streblid bat flies have been documented in Africa [30, 34, 35], mostly in the Democratic Republic of Congo (Central Africa) and South Africa. Some additional records are from Cameroon, Nigeria and East Africa (Kenya, Sudan, Tanzania and Uganda) [30, 34]. However, knowledge on bat flies that parasitize cave-dwelling chiropterans is very scarce, especially in Central Africa.

Recent investigations demonstrated the role of bat flies in the transmission of haemosporidian parasites [36], mirroring the earlier discovery that *Penicillidia fulvida*, an African bat fly species, was involved in *Polychromophilus* transmission in Congo-Brazzaville [15]*.* Moreover, the Palaearctic species *Nycteribia kolenatii* Theodor & Mosconahas is considered to be a vector of *Polychromophilus murinus* Dionisi in cave environments [36, 37]. In Gabon, recent studies on “malaria” parasites infecting cave-dwelling bats revealed that greater long-fingered bats (*Miniopterus inflatus*) are infected by *Polychromophilus melanipherus* [38].

Due to their vector potential, it is therefore crucial to better investigate bat fly diversity, spatial distribution and relationship with their host bat species and to elucidate their vector capacity. To this aim, a longitudinal entomological survey was undertaken to assess the diversity, host preference and implication in parasite transmission of bat flies that infect bats in four caves in Gabon (Central Africa).

## Methods

### Study sites, bat and ectoparasite sampling

**Fig. 1:**
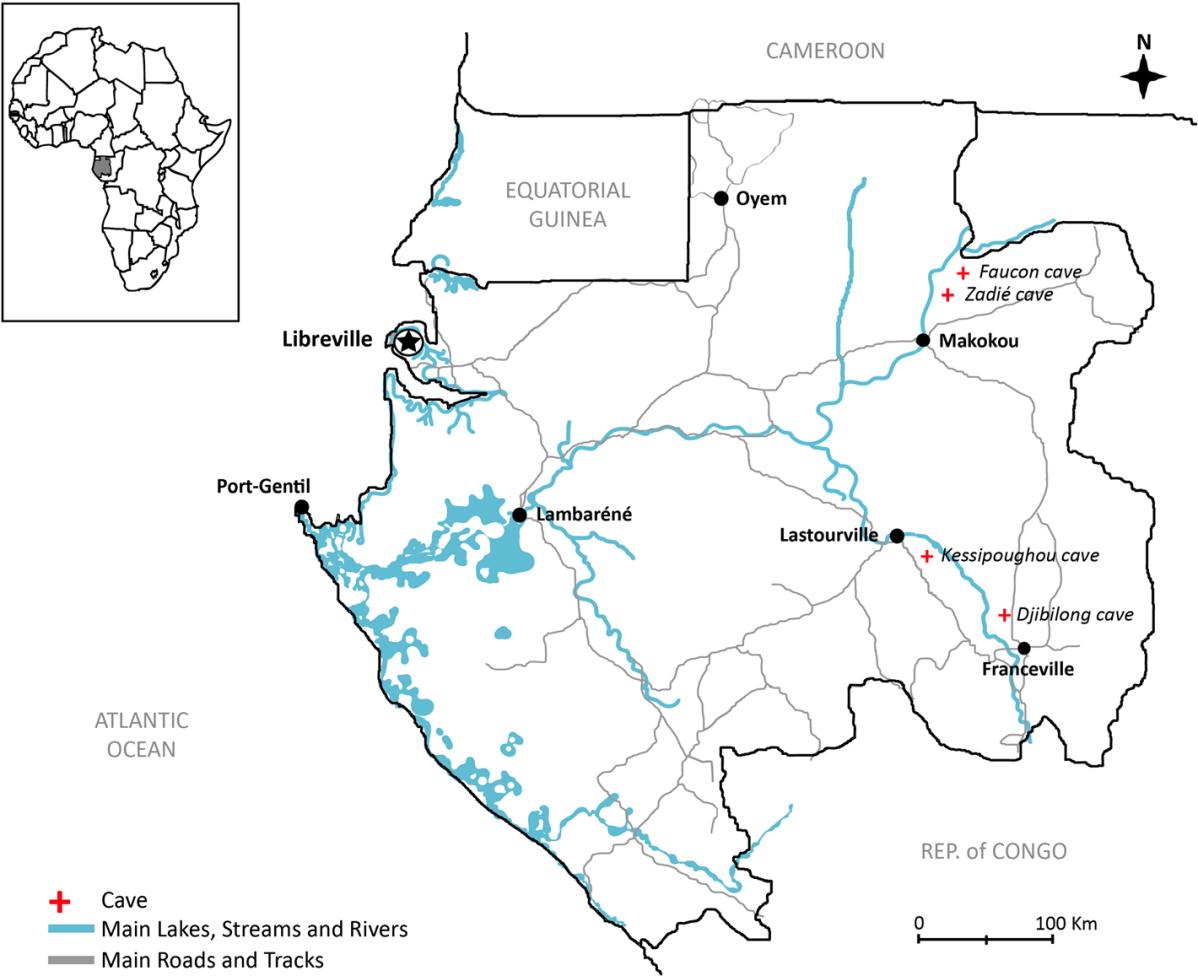
Localization of the four caves in Gabon

Entomological surveys were performed in four caves in Gabon (Fig. 1). The Faucon cave (01.07287N, 13.20739E) and Zadie cave (00.98595N, 13.19745E) are located in the Belinga rainforest (North-East of Gabon) (Fig. 1). The Faucon cave is extremely humid (almost 100 % relative humidity) and has a 4 m high and 6 m wide entrance that leads directly to the main chamber (35 m long, 30 m wide and 10 m high). The main chamber ends in cavities and tunnels that are difficult to access and has a chimney of 1 m in diameter. The Zadie cave is much drier than the Faucon cave and has a large entrance (10 m long by 5 m high) that brings to a funnel-shaped corridor. The corridor leads to the main chamber (900 m^2^ and 5–10 m high). The deeper part of the chamber continues into a secondary, smaller chamber of about 300 m^2^. The Kessipoughou cave (00.86722S, 12.77389E) is located in a forested area near Lastoursville town, and is one of the largest known caves in Gabon. The Kessipoughou cave forms a long tunnel (15–20 m high and variable width reaching about 25–30 m at some places) through which a river runs. There are two entrances shaped by water infiltration and several chambers and galleries. The Djibilong cave (01.36261S, 13.46296E) is located in a mixed savanna-forest area in the South-East of Gabon, near the city of Franceville. The oblique entrance of this cave is about 3 m wide and leads to a large main chamber (40 m wide and 10 m high). At its extremity, the main chamber bifurcates into two secondary chambers with large stagnant ponds created by water infiltration.

Bats were captured monthly from February to April 2011 (Faucon and Zadie caves) and from May 2012 to April 2013 (Kessipoughou and Djibilong caves) using a harp trap (G7 Cave Catcher Harp Trap designed and manufactured by ©Bat Conservation and Management, Inc.) in accordance with the guidelines of the American Society of Mammalogists (http://www.mammalsociety.org/committees/animal-care-and-use). Each month the traps were active from 5:00 pm to 7:00 am, for 2–4 consecutive nights. Bats were identified to the species (or species complex) level using the available identification keys for African bats [39, 40]. All bat flies found on the captured bats were immediately collected with fine forceps and stored in liquid nitrogen for research purposes, including virus screening (not presented in this paper). Bats were released at the cave entrance after bat fly collection. At the International Centre for Medical Research in Franceville (CIRMF), bat fly specimens were taxonomically identified under a binocular microscope, using the identification keys Theodor [30, 35]. Despite the storage in liquid nitrogen, morphological features were well preserved. Once identified, bat fly specimens collected from a single bat individual were grouped in mono-specific pools (i.e. a single bat fly species per pool) containing up to nine fly specimens (i.e. the maximum number of conspecific fly specimens recovered from a single bat). When bats were infested by more than one fly species, the number of pools per bat corresponded to the number of fly species. All pools were stored at -80 °C until analysis.

### Statistical analyses

All statistical analyses were conducted using *R* v*3.0.2* (https://www.r-project.org/). Analysis of variance (ANOVA) was used to compare the bat mean infestation rate and the mean number of bat flies per infested bat according to the bat species, by considering all caves together and separately. To assess the specific association between bat and bat fly species, Poisson generalized linear mixed models (GLMM) were fitted to the number of infested bats relative to the bat fly species, site and month of collection with a log link using the *lme4* package [41]. The interaction between the two response variables, “bats” and “bat flies”, was fitted as a fixed effect in the model. The random effects were “sites” and “months of collection” and both explanatory variables were nested to “bats” species. The significance of the “bats” × “bat flies” interaction was estimated by using the Akaike Information Criterion (AIC) [42] for model selection. AIC changes were evaluated when model parameters were modified (added or removed) [43]. Full-model averages with shrinkage, available in the *MuMIn* package [44], were used for AIC estimation. The best model showed ΔAIC_C_ = 0; however, a variation was considered significant according to the rule ΔAIC_C_ < 2 [45, 46].

### Molecular screening of *Polychromophilus*

The presence of *Polychromophilus* parasites was investigated in single-species bat fly pools from a single host. First, pools were crushed in 300 μl of cold 1× PBS using a ball-mill tissue grinder (Genogrinder 2000, Spex Centripep). Total DNA was extracted from 100 μl of this homogenate with the Qiagen DNeasy Blood and Tissue kit, according to the manufacturer's instructions. A 835 bp fragment of the parasite mitochondrial cytochrome *b* (*cyt b*) gene was amplified using a nested-PCR procedure, as previously described [47], and the GeneAmp PCR System 9700 (Applied Biosystems). PCR products (10 μl) were visualized by electrophoretic migration on 1.5 % agarose gels in 1× TBE buffer, and positive amplicons were sent to Beckman Coulter Genomics (France) for bidirectional sequencing using original primers.

### Sequence analysis and the Maximum Likelihood Estimates of Infection Rate (MLE-IR)

Geneious 7.0.6 (Biomatters, www.geneious.com) was used for sequence analysis (correction, alignment, mapping against reference sequences from GenBank). For phylogenetic analyses, maximum likelihood (ML) trees were constructed based on the alignment of our 835 bp sequences with a subset of previously published malaria parasite *cyt b* sequences (see Additional file 1: Table S1) for genus assignment. Conversely, for species assignment the amplified sequences were aligned with previously published *Polychromophilus* spp. *cyt b* sequences, but only a 314 bp long region was common to all these sequences and was used for alignment and tree construction. The best-fitting ML model under the Akaike Information Criterion was GTR (General Time Reversible) + Γ (Gamma distribution) + I (Invariable sites’ distribution), as identified by Model Test [48]. The highest-likelihood DNA trees and corresponding bootstrap supporting values were obtained with PhyML (freely available at the ATGC bioinformatics platform http://www.atgc-montpellier.fr/) using NNI (Nearest Neighbor Interchange) + SPR (Subtree Pruning Regrafting) branch swapping and 100 bootstrap replicates [49]. Infection rates (percentage of infected pools relative to the total number of screened pools) were calculated and the Maximum Likelihood Estimates of bat fly infection rates (MLE-IR) were computed with 99 % of confidence level using the MLE-IR program [50]. MLE-IR is a corrected estimate of the infection rate that takes into account the number of bat fly individuals present in each pool. For example, the MLE of a given bat fly species has to be understood as the percentage of infected flies relative to all screened individuals of that bat fly species.

## Results

### Bat diversity and abundance

In total, 1,154 bats were captured and were assigned to five species/complexes: *Miniopterus inflatus* (*n* = 354), *Hipposideros gigas* (*n* = 317)*, Hipposideros caffer* complex (*n* = 285), *Rousettus aegyptiacus* (*n* = 157) and *Coleura afra* (*n* = 41) (Table 1). Important qualitative (i.e. trapped bat species) and quantitative (i.e., trapped bat number) variations were observed between caves and only bats belonging to the *H. caffer* complex were captured in all four caves. Independently of seasonality, most *H. caffer* complex and *M. inflatus* specimens were captured in Kessipoughou and Djibilong caves, whereas *H. gigas* and *R. aegyptiacus* were more numerous in Kessipoughou and Zadie caves, respectively. *Coleura afra* was only captured in Faucon cave (Table 1). Bat capture rates (average number of bats captured per night) during the entire study period varied significantly among caves (ANOVA, *F*_(3,56)_ = 27.4, *P* < 0.0001), with mean nightly catches of 51.0, 45.5, 16.0 and 10.9 bats in Zadie, Faucon, Kessipoughou and Djibilong cave, respectively (Additional file 1: Table S2). Finally, although bats were sampled throughout the year in both Kessipoughou and Djibilong caves, their numbers significantly varied seasonally in Kessipoughou (*χ*^2^ = 152.9, *df* = 11, *P* < 0.0001) and in Djibilong cave (*χ*^2^ = 267.3, *df* = 11, *P* < 0.0001) (Additional file 1: Table S3).

**Table 1:** Distribution of bat flies in the different caves and bat species. The number of flies of a given species found on each bat species and in each cave is shown. Numbers between brackets refer to the number of bats infested by a given bat fly species. For each cave, the sum of all infested bats by a given bat fly species is not necessarily equal to the total number of infested bats due to the co-infestation by different bat fly species of one bat individual.

	Bat species					
	*C.a*	*H.c*	*H.g*	*M.i*	*R.a*	All bat species
Faucon cave						
Total bat number	41	41	31	71	0	184
Total infested bat number	4	2	0	62	–	68
*E. africana*	0	2 (1)	–	0	–	2 (1)
*N. schmidlii scotti*	1 (1)	0	–	134 (55)	–	135 (56)
*P. fulvida*	4 (3)	1 (1)	–	25 (20)	–	30 (24)
Zadie cave						
Total bat number	0	66	77	0	111	254
Total infested bat number	–	1	0	–	31	32
* E. africana*	–	1 (1)	–	–	59 (31)	60 (32)
Kessipoughou cave						
Total bat number	0	90	209	126	40	465
Total infested bat number	–	41	50	84	29	204
* E. africana*	–	0	4 (3)	0	91 (26)	95 (29)
*N. schmidlii scotti*	–	8 (5)	4 (3)	187 (80)	11 (3)	210 (91)
*P. fulvida*	–	0	1 (1)	16 (13)	0	17 (14)
*R. huberi*	–	89 (35)	6 (6)	0	0	95 (41)
*B. allaudi*	–	2 (2)	53 (39)	2 (2)	1(1)	58 (44)
Djibilong cave						
Total bat number	0	88	0	157	6	251
Total infested bat number	–	12	–	120	3	135
*E. africana*	–	0	–	3 (1)	7 (2)	10 (3)
*N. schmidlii scotti*	–	2 (2)	–	306 (114)	2 (1)	310 (117)
*P. fulvida*	–	0	–	24 (17)	0	24 (17)
*R. huberi*	–	15 (10)	–	0	0	15 (10)
*B. allaudi*	–	0	–	2 (1)	0	2 (1)
All caves						
Total bat number	41	285	317	354	157	1154
Total infested bat number	4	56	50	266	63	439
*E. africana*	0	3 (2)	4 (3)	3 (1)	157 (59)	167 (65)
*N. schmidlii scotti*	1 (1)	10 (7)	4 (3)	627 (249)	13 (4)	655 (264)
*P. fulvida*	4 (3)	1 (1)	1 (1)	65 (50)	0	71 (55)
*R. huberi* group	0	104 (45)	6 (6)	0	0	110 (51)
*B. allaudi*	0	2 (2)	53 (39)	4 (3)	1 (1)	60 (45)

*Abbreviations*: *C.a* *Coleura afra*, *H.c* *Hipposideros caffer* complex, *H.g* *Hipposideros gigas*; *M.i* *Miniopterus inflatus*, *R.a* *Rousettus aegyptiacus*

### Bat fly diversity and bat infestation

Bat examination allowed collecting 1,063 bat fly specimens (Table 1) that belonged to the families Streblidae (16.6 %) and Nycteribiidae (83.4 %). Among nycteribiid flies, three species were identified: *Nycteribia schmidlii scotti* (73.3 %), *Eucampsipoda africana* (18.7 %) and *Penicilidia fulvida* (8.0 %). Three streblid fly species were detected: *Brachytarsina allaudi* (35.3 %), *Raymondia huberi* and a *Raymondia* species known as sp. A [30]. The two last species both belong to the *Raymondia huberi* group and together accounted for 64.7 % of all streblid flies (Table 1).

Bat flies were found on 439 (38.0 %) of the 1,154 collected bats. When considering all caves together, the mean infestation rate varied significantly according to the bat species (ANOVA *F*_(4,75)_ = 13.15, *P* < 0.0001) (Table 2)*.* Species-specific differences in bat infestation rate were confirmed also when each cave was considered on its own (Table 2). The infestation rates for a given bat species were similar in all caves. Only the *H. caffer* complex infestation rates in the Faucon and Zadie caves and the *R. aegyptiacus* infestation rate in Zadie cave were much lower than in the other caves (Table 2). The mean number of bat flies collected per infested bat varied significantly according to the bat species (ANOVA *F*_(4,1149)_ = 88.28, *P* < 0.0001) (Table 3) both when all caves or each single cave were considered. Moreover, the mean number of bat flies per infested bat of a given species was similar in all caves (Table 3).

**Table 2:** Comparison of the mean infestation rates (%) in the different bat species

	*M.i*	*H.c*	*H.g*	*R.a*	*C.a*	ANOVA		
						*F*	*df*	*P*
All caves	74.21 ± 3.19	26.03 ± 6.61	23.97 ± 8.44	66.52 ± 9.72	4.43 ± 4.43	13.15	75	< 0.0001
Kessipoughou	69.74 ± 4.30	36.75 ± 11.07	38.36 ± 11.35	79.72 ± 11.81	na	4.65	33	0.008
Djibilong	78.14 ± 4.77	30.13 ± 11.57	na	62.50 ± 23.93	na	5.96	20	0.009
Faucon	76.06 ± 13.38	4.43 ± 4.43	0	na	4.43 ± 4.43	24.54	8	< 0.0001
Zadie	na	2.50 ± 2.50	0	27.90 ± 1.45	na	135.80	5	< 0.0001

*Abbreviations*: *C.a* *Coleura afra*, *H.c* *Hipposideros caffer* complex, *H.g* *Hipposideros gigas*, *M.i* *Miniopterus inflatus*, *R.a* *Rousettus aegyptiacus*, *ANOVA*, analysis of variance, *F* ANOVA F-statistic, *df* degrees of freedom, *P* *P*-value, *na*, not applicable (i.e. missing data)

**Table 3:** Mean number of bat flies per infested bat in the different bat species

	*M.i*	*H.c*	*H.g*	*R.a*	*C.a*	ANOVA		
						*F*	*df*	*P*
All caves	1.97 ± 0.09	0.42 ± 0.06	0.21 ± 0.03	1.08 ± 0.13	0.12 ± 0.06	88.28	1149	< 0.0001
Kessipoughou	1.62 ± 0.15	1.10 ± 0.17	0.32 ± 0.04	2.57 ± 0.37	na	39.51	461	< 0.0001
Djibilong	2.13 ± 0.15	0.19 ± 0.06	na	1.50 ± 0.71	na	40.55	248	< 0.0001
Faucon	2.23 ± 0.19	0.07 ± 0.05	0	na	0.12 ± 0.06	52.20	180	< 0.0001
Zadie	na	0.01 ± 0.01	0	0.53 ± 0.08	na	21.89	251	< 0.0001

*Abbreviations*: *C.a* *Coleura afra*, *H.c* *Hipposideros caffer *complex, *H.g* *Hipposideros gigas*, *M.i* *Miniopterus inflatus*, *R.a* *Rousettus aegyptiacus*, *ANOVA*, analysis of variance, *F* ANOVA F-statistic, *df* degrees of freedom, *P* *P*-value, *na*, not applicable (i.e. missing data)

To assess whether bat fly species were randomly distributed among bat species, generalized linear mixed models (GLMM) were used. Due to the low number of infested individuals (*n* = 4), *C. afra* bats were omitted from the overall analysis. The results revealed a strong association effect between bats and bat flies, which represent the most important parameters in the model (see Additional file 1: Table S4). *Miniopterus inflatus* was commonly and significantly infested by the bat fly species *N. s. scotti* (*Z*-value = 3.17, *P* < 0.0001) and *P. fulvida* (*Z*-value = 2.55, *P* = 0.01)*.* On the other hand, *E. africana* flies (the other nycteribiid species) were preferentially associated with *R. aegyptiacus* (*Z*-value = 2.84, *P* < 0.0001). Similarly, species of the *R. huberi* group and *B. allaudi* were preferentially associated with bats belonging to the *H. caffer* complex and *H. gigas*, respectively. All five bat fly taxa were occasionally found on non-common hosts (up to 13 % for *B. allaudi*), suggesting that the preferential host-bat fly associations, even if strong, are not strict. Some of these occasional associations could result from accidental transfers of flies to non-usual hosts, including during the bat collection process (i.e. different species retained together into the catch bag of the harp trap). However, two bat fly species (*E. africana* in Faucon cave and *B. allaudi* in Djibilong cave) were recovered on non-preferred hosts in the absence of their preferred hosts, suggesting that these species can occasionally infest several bat species. Different bat fly species, mostly Nycteribiidae*-*Nycteribiidae associations (*n* = 36 *N. schmidlii scotti* with *P. fulvida*) on *M. inflatus* bats, were identified in 41 bats (9.3 % of all infested bats).

**Fig. 2:**
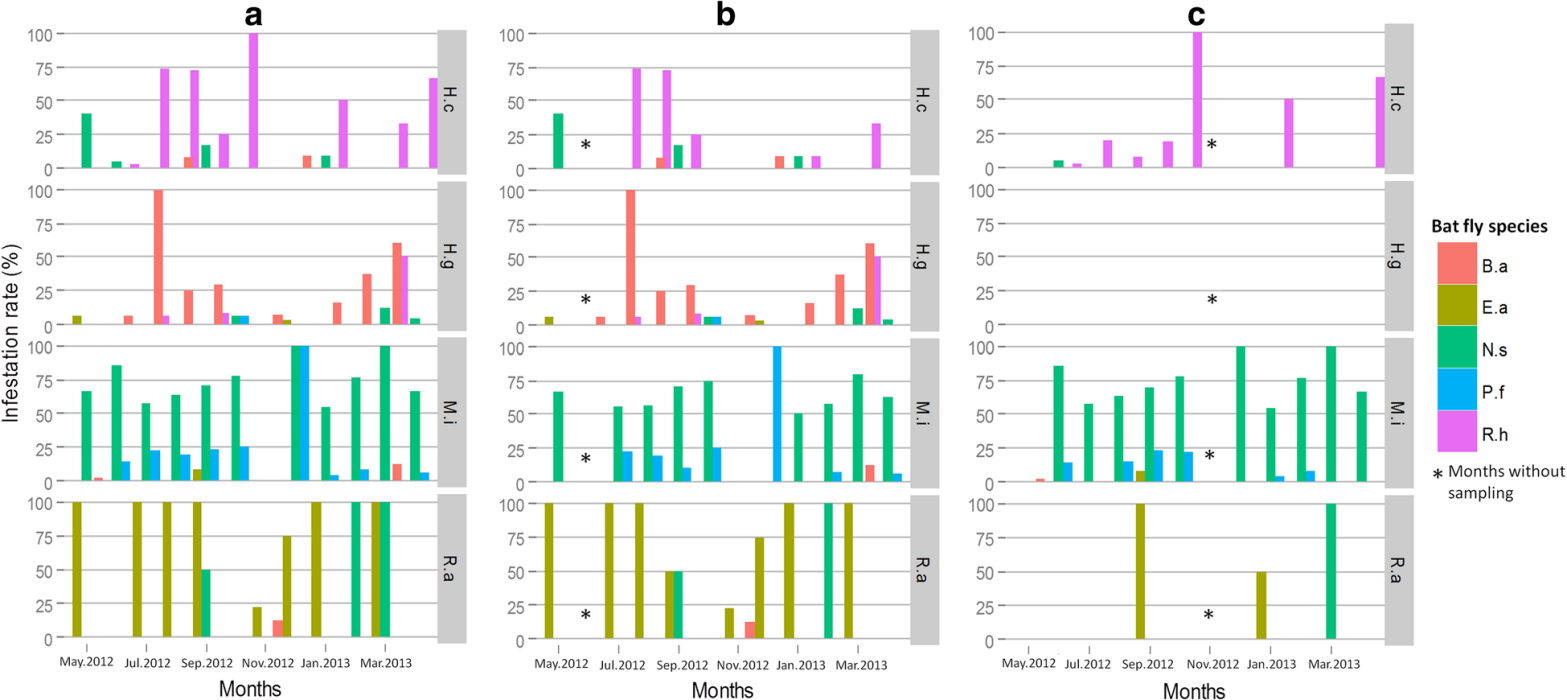
Temporal variation of bat infestation rates in Kessipoughou and Djibilong caves. **a** Both caves. **b** Kessipoughou cave. **c** Djibilong cave. Infestation rate indicates the percentage of bats infested by at least one bat fly individual relative to the total number of collected bats. *Abbreviations*: B.a., *Brachytarsina allaudi*; E.a., *Eucampsipoda africana*; N.s., *Nycteribia schmidlii scotti*; P.f., *Penicillicidia fulvida*; R.h., *Raymondia huberi* group. Star indicates month without sampling

Temporal variations of the infestation rate were assessed by analyzing data only from Kessipoughou and Djibilong caves where sampling was done longitudinally. The infestation rate was calculated for each month, each bat species and each bat fly species (Fig. 2). When both caves were considered together, infestation rates varied significantly in time for *H. caffer* complex (*χ*^2^ = 299.4, *df* = 9, *P* < 0.0001), *H. gigas* (*χ*^2^ = 304.9, *df* = 9, *P* < 0.0001), *M. inflatus* (*χ*^2^ = 32.9, *df* = 10, *P* < 0.0001) and *R. aegyptiacus* (*χ*^2^ = 249.7, *df* = 10, *P* < 0.0001) (Additional file 1: Table S5 and Fig. 2a). However, in both Kessipoughou and Djibilong caves, *M. inflatus* bats seemed to be continuously infested by *N. schmidlii scotti* and *P. fulvida*, whereas *H. caffer* complex, *H. gigas* and *R. aegyptiacus* bats were more frequently infected by *R. huberi* group, *B. allaudi* and *E. africana* flies, respectively (Fig. 2a). When considering the two caves separately, infestation rates still significantly changed throughout the year (Additional file 1: Table S5 and Fig. 2b, c). However, infested *H. gigas* specimens were mostly collected in Kessipoughou. Although infestation rates varied in both caves throughout the year, the mean number of flies per infested bat remained stable (all ANOVA *P-*values > 0.05) over the year, whatever the bat species (Additional file 1: Table S6).

### Molecular screening and dynamics of *P. melanipherus* infection

**Fig. 3:**
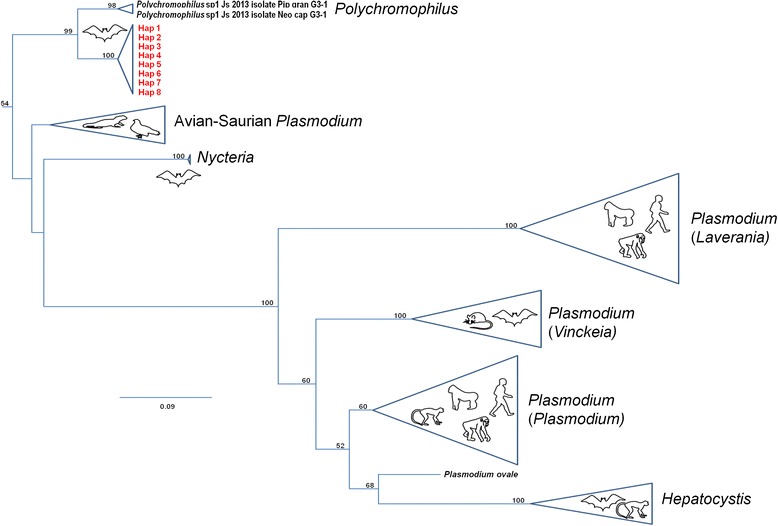
Phylogenetic position of bat fly-infecting parasites for genus assignment. Maximum likelihood sub-tree of the species of Haemosporidia obtained from the alignment analysis of 835 bp *cyt b* sequences. *Parahaemoproteus vireonis* and *Haemoproteus columbae* were used to root the tree. See Methods section and Additional file 1: Table S1 for details and GenBank accession numbers of the different sequences included in the phylogeny. The bootstrap values are indicated at each node, when > 0.5

**Fig. 4:**
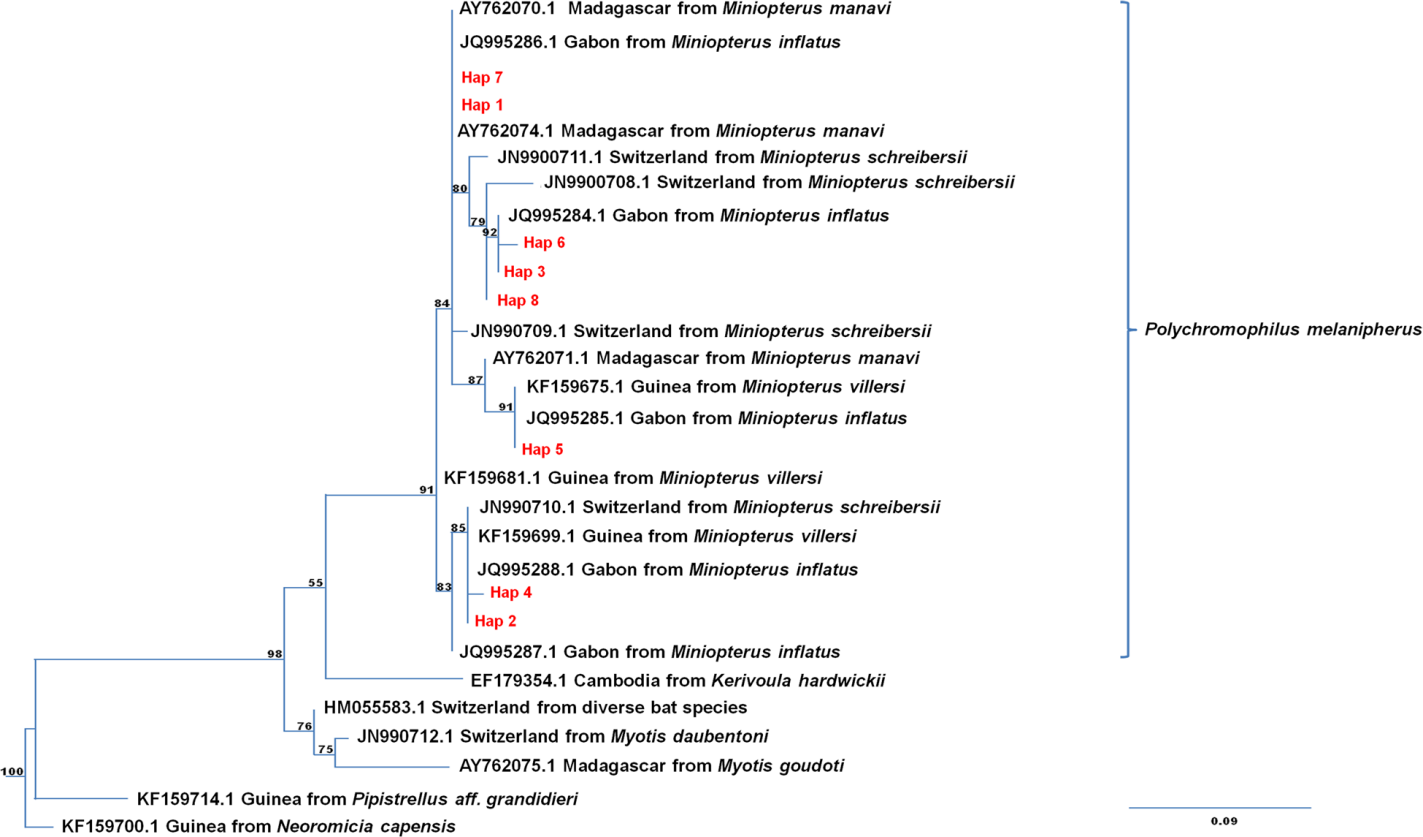
Phylogenetic position of bat fly-infecting parasites for species assignment. Maximum likelihood sub-tree of *Polychromophilus* species obtained from the alignment analysis of 314 bp *cyt b* sequences. *Nycteria* sp*.* parasites infecting bats were used to root the tree. See Methods section and Additional file 1: Table S1 for details and GenBank accession numbers of the different sequences included in the phylogeny. The bootstrap values are indicated at each node, when > 0.5

Infection by haemosporidian parasites was assessed in the 416 bat fly pools that originated from 375 infested bats captured in Faucon (*n* = 37), Zadie (*n* = 11), Kessipoughou (*n* = 221) and Djibilong cave (*n* = 147). Haemosporidian parasites were detected in 75 pools from three of the four caves: 5 from Faucon (13.51 %), 27 from Kessipoughou (12.21 %) and 43 from Djibilong cave (29.25 %). Based on mtDNA *cyt b* sequencing and phylogenetical analysis, all haemosporidian parasites were identified as *Polychromophilus melanipherus* (Figs. 3 and 4).

**Fig. 5:**
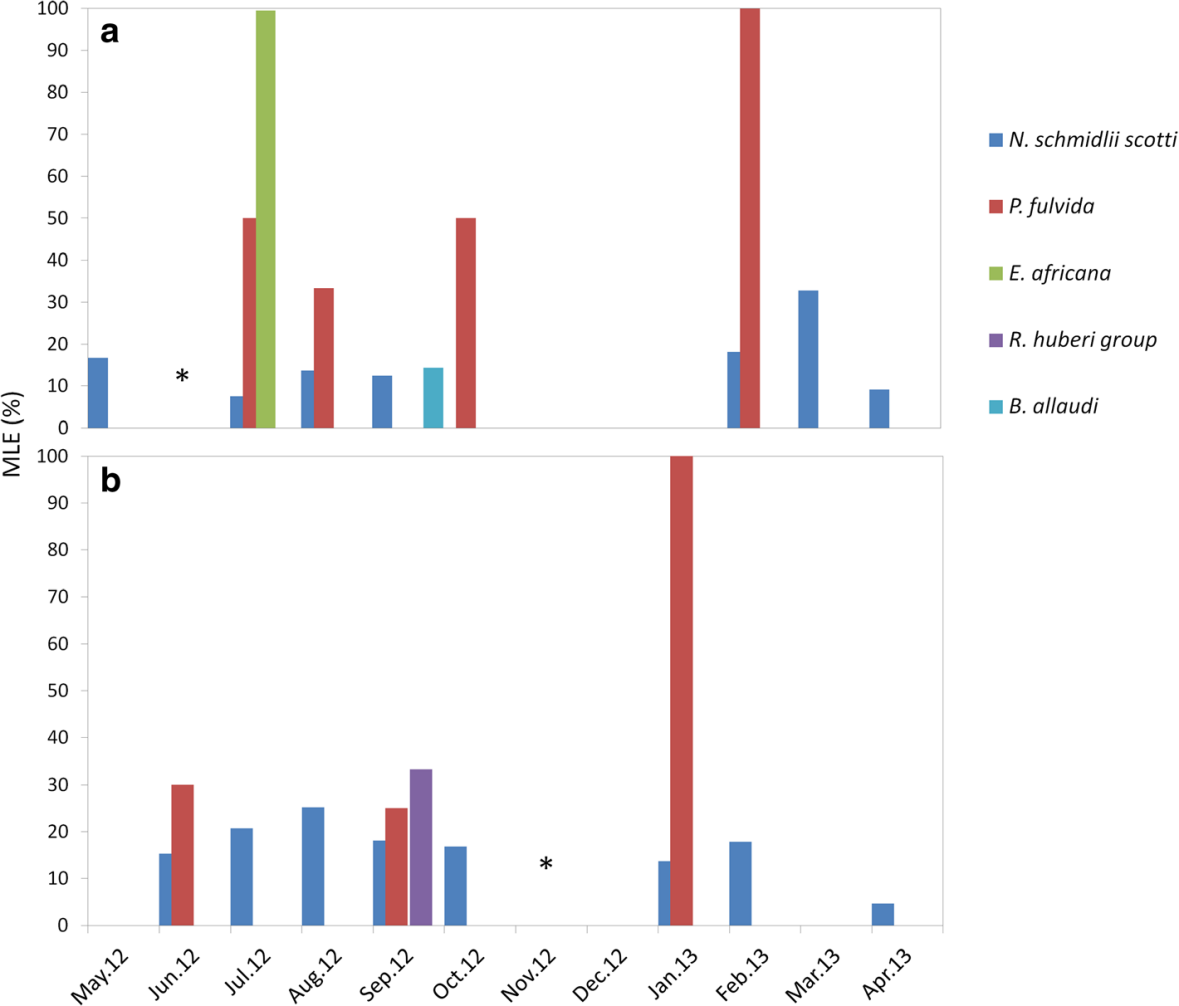
*Polychromophilus melanipherus* Maximum Likelihood Estimates of the infection rate (MLE) dynamics in Kessipoughou and Djibilong caves. **a** Kessipoughou cave. **b** Djibilong cave. Star indicates month without sampling

The MLE-IR values (see Additional file 1: Table S7) indicated that *P. melanipherus* was mainly detected in *N. schmidlii scotti* and *P. fulvida* bat flies (both nycteribiid species) and less frequently in *E. africana, R. huberi* group and *B. allaudi*. Most (*n* = 71) infected bat flies pools were from *M. inflatus* bats (63 pools of *N. schmidlii scotti*, 8 pools of *P. fulvida*), and the other four positive pools included flies collected from *C. afra* (one *N. schmidlii scotti* pool), *H caffer* complex (one *B. allaudi* and one *R. huberi* group pool) and *R. aegyptiacus* (one *E. africana* pool) bats*.* Among all bat fly taxa, infected *N. s. scotti* specimens were detected almost each month in both Kessipoughou (Fig. 5a) and Djibilong (Fig. 5b) cave, whereas infected *P. fulvida* bats peaked three to four times during the year. Conversely, infected *E. africana*, *B. allaudi* (in Kessipoughou cave) and *R. huberi* group animals (in Djibilong cave) were only found occasionally.

Analysis of the *P. melanipherus cyt b* sequences identified 18 segregation sites that defined eight distinct haplotypes (Hap1 to Hap8; see Additional file 1: Table S8). Hap1 (46.7 %), Hap2 (34.7 %) and Hap3 (12 %) were the most frequent, whereas Hap4 to 8 accounted for less than 2 %/each. Hap1 and 2 were similarly detected in all caves, whereas Hap3 was restricted to Kessipoughou and Djibilong caves (Additional file 2: Fig. S1). Hap5 and 7 were found only in Kessipoughou cave, and Hap4, 6 and 8 were only detected in Djibilong cave (Additional file 2: Fig. S1).

## Discussion

### Bat fly diversity and host preference

The Nycteribiidae and Streblidae fauna of Gabon has been poorly studied, with only a single species (*P. fulvida*) reported so far [51]. This study identified five new bat fly species found on cave-dwelling bats: two Nycteribiidae (*N. schmidlii scotti* and *E. africana)* and three Streblidae (*B. allaudi* and two species of the *R. huberi* group).

This is the first record of *E. africana* in Central Africa. This species was previously reported in West (Sierra Leone), East (Kenya) and South Africa (Botswana and the Republic of South Africa), where it infests mainly *R. aegyptiacus* and more rarely *Eidolon helvum* bats [30]. The two other nycteribiid species (*N. schmidlii scotti* and *P. fulvida)* have been already described in Central (Cameroon and Congo-Brazzaville), West (Nigeria), East (Kenya, Sudan) and South Africa (Republic of South Africa, Zimbabwe), where they infest many cave-dwelling bat species, mainly of the genus *Miniopterus* (both *N. schmidlii scotti* and *P. fulvida*) and also of the genera *Nycteris* (*N. schmidlii scotti*), *Pipistrellus* (*N. schmidlii scotti*), *Eptesicus* (*Nycteribia* sp.), *Eidolon* (*P. fulvida*), *Vespertilio* (*P. fulvida*), *Rhinolophus* and *Hipposideros* (both *N. schmidlii scotti* and *P. fulvida*) [14, 15, 30, 34]. The three streblid species detected in this study (*B. allaudi* and the two species of the *R. huberi* group) have never been reported in Central Africa, but have been documented in East Africa, where they infest mainly bats of the genus *Hipposideros,* and less frequently of the genera *Cardioderma, Triaenops* (*R. huberi* group) and *Epomophorus* (*B. allaudi*) [35].

All five bat fly species were found in the Kessipoughou and Djibilong caves, but not in the Faucon and Zadie caves. This could be explained by sampling variations or by qualitative and quantitative differences in bat species assemblages in each cave. Five bat species were captured, all of which were previously reported in Gabon [52] and are known to roost in cave-dwelling habitats across the African continent [4]. However, bat communities can vary according to the cave type, and the presence/absence of a particular bat species probably depends on the habitat suitability inside and around each cave. For example, *R. aegyptiacus* and *M. inflatus* were absent in Faucon and Zadie cave respectively, whereas *C. afra* was only captured in Faucon cave. The fruit bat *R. aegyptiacus* generally selects ecological environments with abundant fruit trees and dark roosts [53]. Therefore, Faucon cave, with its large entrance and chimney bringing light deep inside the main chamber is less suitable for *R. aegyptiacus*. Bats of the genus  *Miniopterus* prefer moister caves [54, 55], which probably explains their presence in Faucon cave (a very moist cave), and their absence in Zadie cave which is much dryer (personal observation).

Modulation of the bat fly community composition by the bat community in a given environment requires the existence of some degrees of bat fly preference for host bat species. Bat flies exhibit a high degree of host-specificity [56] and most species infest only a single bat species or phylogenetically related bats species [31, 56, 57]. This host specialization is the result of long co-evolutionary processes that led to morphological, behavioral and physiological adaptations of bat flies and also to the development of immunocompatibility between bat flies and their primary host and to the frequent vertical transfer of bat flies from adult bats to their offspring [29, 56]. Here, we found that each bat fly species identified in Gabon caves is preferentially associated with a single main host. *Nycteribia schmidlii scotti* and *P. fulvida* were both preferentially associated with *M. inflatus*; *E. africana* with *R. aegyptiacus*; the *R. huberi* group with *H. caffer* complex and *B. allaudi* with *H. gigas.* On the other hand, *C. afra* was infested by *N. schmidlii scotti* and *P. fulvida*, but no preferential association with either fly species could be identified because of the low number of parasitized specimens. However, a previous study reported infestation of *C. afra* by *P. fulvida* in other regions of the world [58]. Despite the apparent specificity between bats and bat flies, we recorded some associations involving one or more non-primary hosts that could have originated from natural infestation or accidental transfers. Accidental transfers from the primary to an accidental host can occur during bat sampling [56], especially through contact between bat species in the bag of the harp trap. Nevertheless this route can be ruled out in Faucon and Djibilong caves where *E. africana* and *B. allaudi* were recovered on accidental hosts in the absence of their respective primary host. Complementary analysis, including blood meal identification, should be done to determine whether accidental hosts can really serve as secondary hosts.

### Temporal heterogeneity of bat infestation rate and abundance

The main consequence of this host preference is the interdependence of the bat and bat fly communities in space and time. Indeed, although the mean number of bat flies per bat remained stable over time, the infestation rates at the Kessipoughou and Djibilong caves significantly changed during the year. This suggests that the infestation rate (i.e. the way bat flies spread within bat communities) is a better parameter to assess bat fly parasitism. The infestation rate variations seemed to mirror the size changes in the collected bat populations throughout the year. Additional and more accurate investigations on the abundance of bat fly species relative to climatic conditions and intrinsic bat parameters (e.g. ecology, physiology) should be performed. Nevertheless it is obvious that infestation rate variations can significantly influence the transmission dynamics of bat fly-borne pathogens, particularly *P. melanipherus*.

Bats were collected throughout the year in Kessipoughou and Djibilong caves; however significant variations in bat abundance were observed according to the sampling month. Similar observations were reported for *H. caffer* complex bats in Uganda [59] and for other cave-dwelling bat species across the world [60], where a decrease of bat trapping was recorded during the rainy season, which negatively affects bat exit. Alternatively, the decrease in the number of captured bats could indicate a reduction in the bat populations inside the caves due to migratory events [61–63] or behavioral changes related to parturition, which influences the exit, especially of female bats that rest in nurseries located deeper in caves [4]. In Afro-tropical regions, particularly in Gabon, parturition events occur mainly in March (e.g. *H. caffer* complex) and in October (most cave-dwelling bat species) [59, 61, 64] and a sampling reduction was observed in these months.

### Detection, spatial and temporal dynamics of *P. melanipherus* infection

The role of some nycteribiid species as vector of *Polychromophilus* parasites has previously been demonstrated in cave ecosystems of tropical [15] and temperate areas [36, 37, 65]. Our molecular screening provided evidence for the presence of *P. melanipherus* in *N. s scotti* and *P. fulvida* bat flies. The respective infection rates strongly suggest that both species are involved in *P. melanipherus* transmission in Gabon. This hypothesis is strengthened by a previous parasitological study in the Faucon cave showing a high prevalence of *P. melanipherus* in *M. inflatus*, a bat we found to be preferentially infested by *N. s scotti* and *P. fulvida* [38]. This study also demonstrated that *P. melanipherus* infection was restricted to *M. inflatus* in accordance with the finding that the range of *P. melanipherus* hosts is limited to bats belonging to the family Miniopteridae [66]. Unexpectedly, we detected *P. melanipherus* infections also in a small number of bat fly pools (one pool for each taxon: *N. schmidlii scotti, E. africana, B. allaudi* and *R. huberi* group) collected on non-Miniopteridae bats (*H. caffer* complex, *R. aegyptiacus* and *C. afra* from the families Hipposideridae, Pteropodidae and Emballonuridae). This implies that, given the strong specificity of *P. melanipherus* for Miniopteridae, the infected *E. africana, B. allaudi* and *R. huberi* group bat fly specimens acquired the parasite during a blood meal on *M. inflatus,* which is not their primary host. Therefore, host switching leading to blood-feeding really occurs and bat flies can occasionally infest non-primary hosts.

This is the first time that *P. melanipherus* is identified in *N. s. scotti*, *E. africana*, *B. allaudi* and *R. huberi* group bat flies. However, its detection in the whole body of these bat flies is not enough to definitively prove their role in *P. melanipherus* transmission. Further studies, including the detection of *Polychromophilus* parasites at the infecting stage in salivary glands, are needed to confirm their potential role as vectors. To date, streblid flies have never been found to transmit haemosporidian parasites. Nevertheless, a recent study revealed the presence of infecting stages of a bat malaria parasite (*Vetufebrus ovatus*) in a fossilized specimen of streblid fly [67].

Our results indicate that there is no significant difference in bat fly *P. melanipherus* infection rates between caves, suggesting quite similar spatial patterns of *P. melanipherus* transmission. However, *P. melanipherus* infection rates varied in time in the Kessipoughou and Djibilong caves. This could mirror the transmission dynamics of this malaria parasite in these caves that seems to follow the temporal variations in bat infestation. The long-term dynamics of haemosporidian infections in bat flies and bat colonies are poorly documented in tropical areas. However, a previous study in Faucon cave showed that the highest *P. melanipherus* infection rates coincide with the months of heavy bat infestation by bat flies [38].

## Conclusion

The present work describes the cave-dwelling bat fly fauna of Gabon and highlights some host preference trends for each reported bat fly species. Bat fly assemblage in caves was related to the bat population composition and the infestation rates varied during the year. Our investigation brings insights into *N. schmidlii scotti* and *P. fulvida* involvement in the transmission of *Polychromophilus* parasites.

## Abbreviations

AIC, akaike information criterion; ANOVA, analysis of variance; bp, base pair; *cyt b*, cytochrome *b* gene; df, degrees of freedom; DNA, deoxyribonucleic acid; *F*, ANOVA F-statistic; GLMM, generalized linear mixed models; GTR, general time reversible; ML, maximum likelihood; MLE-IR, maximum likelihood estimates of infection rates; mtDNA, mitochondrial deoxyribonucleic acid; na, not applicable; NNI, nearest neighbor interchange; P, *P*-value; PBS, Phosphate-buffered saline; PCR, polymerase chain reaction; SPR, subtree pruning regrafting; TBE, tris borate EDTA

## Additional files

Additional file 1: Tables S1 to S8. (DOCX 62 kb) Additional file 2: Figure S1. Polychromophilus melanipherus haplotype distribution. Pie charts showing the *P. melanipherus* haplotype distribution in the Faucon, Kessipoughou and Djibilong caves. No *P. melanipherus* infection was detected in bat flies collected from bats captured in Zadie cave. (TIF 845 kb)
