# Supplementary material for: Bat flies (Diptera: Nycteribiidae and Streblidae) infesting cave-dwelling bats in Gabon: diversity, dynamics and potential role in Polychromophilus melanipherus transmission
Source: Parasit Vectors. 2016 Jun 10;9:333. doi: 10.1186/s13071-016-1625-z (PMC4902993; doi:10.1186/s13071-016-1625-z)
Supplement: Additional file 1: — Tables S1 to S8. (DOCX 62 kb) [file 13071_2016_1625_MOESM1_ESM.docx]

**Table S1:** Published sequences of the different malaria parasites used for phylogenetic analysis.

| **Parasite** | **Host** | **Country** | **GenBank Accession Number** | **additional information** | **Phylogenetical Analysis** |
| --- | --- | --- | --- | --- | --- |
| *Plasmodium gallinaceum* | Birds | Viet Nam | AY099029 | - | Analysis1 (835 bp) |
| *Plasmodium relictum* | Birds | United States of America | AY733090 | - | Analysis1 (835 bp) |
| *Plasmodium floridense* | Lizards |  | EF079654 | - | Analysis1 (835 bp) |
| *Plasmodium minuoviridae* | Lizards | Papua New Guinea | EU834703 | - | Analysis1 (835 bp) |
| *Plasmodium giganteum* | Lizards | Ghana | AY099053 | - | Analysis1 (835 bp) |
| *Plasmodium berghei* | Rodents | Democratic Republic of Congo | DQ414645 | - | Analysis1 (835 bp) |
| *Plasmodium chabaudi* | Rodents | Central African Republic | DQ414649 | - | Analysis1 (835 bp) |
| *Plasmodium vinckei* | Rodents | Democratic Republic of Congo | DQ414651 | - | Analysis1 (835 bp) |
| *Plasmodium yoelii* | Rodents | Central African Republic | DQ414660 | - | Analysis1 (835 bp) |
| *Plasmodium sp* | Bats | Guinea | KF159671 | - | Analysis1 (835 bp) |
| *Plasmodium sp* | Bats | Liberia | KF159710 | - | Analysis1 (835 bp) |
| *Plasmodium knowlesi* | Asian Monkeys, Humans |  | AY722797 | - | Analysis1 (835 bp) |
| *Plasmodium fragile* | Asian Monkeys |  | AY722799 | - | Analysis1 (835 bp) |
| *Plasmodium cynomolgi* | Asian Monkeys |  | AY800108 | - | Analysis1 (835 bp) |
| *Plasmodium inui* | Asian Monkeys |  | AB354572 | - | Analysis1 (835 bp) |
| *Plasmodium hylobati* | Gibbons |  | AB354573 | - | Analysis1 (835 bp) |
| *Plasmodium simiovale* | Asian Monkeys |  | AB434920 | - | Analysis1 (835 bp) |
| *Plasmodium coatneyi* | Asian Monkeys |  | AB354575 | - | Analysis1 (835 bp) |
| *Plasmodium vivax* | Gorillas, Chimpanzees, Humans |  | NC007243 | - | Analysis1 (835 bp) |
| *Plasmodium simium* | New World Monkeys |  | NC007233 | - | Analysis1 (835 bp) |
| *Plasmodium sp* | African Monkeys |  | AY800112 | - | Analysis1 (835 bp) |
| *Plasmodium gonderi* | African Monkeys |  | AB434918 | - | Analysis1 (835 bp) |
| *Plasmodium malariae* | Chimpanzees, Humans |  | AB354570 | - | Analysis1 (835 bp) |
| *Plasmodium ovale* | Chimpanzees, Humans |  | AB354571 | - | Analysis1 (835 bp) |
| *Plasmodium adleri* | Gorillas | Cameroon | HM235284 | - | Analysis1 (835 bp) |
| *Plasmodium blacklocki* | Gorillas | Democratic Republic of Congo | HM235376 | - | Analysis1 (835 bp) |
| *Plasmodium gaboni* | Chimpanzees | Gabon | FJ895307 | - | Analysis1 (835 bp) |
| *Plasmodium billbrayi* | Chimpanzees | Democratic Republic of Congo | GQ355468 | - | Analysis1 (835 bp) |
| *Plasmodium billcollinsi* | Chimpanzees | Democratic Republic of Congo | GQ355479 | - | Analysis1 (835 bp) |
| *Plasmodium reichenowi* | Chimpanzees |  | NC002235 | - | Analysis1 (835 bp) |
| *Plasmodium praefaciparum* | Gorillas | Democratic Republic of Congo | HM235308 | - | Analysis1 (835 bp) |
| *Plasmodium praefalciparum* | Gorillas | Cameroon | HM235367 | - | Analysis1 (835 bp) |
| *Plasmodium falciparum* | African Monkeys | Gabon | JF923762 | - | Analysis1 (835 bp) |
| *Plasmodium falciparum* | Gorillas | Gabon | JF923761 | - | Analysis1 (835 bp) |
| *Plasmodium falciparum* | Chimpanzees | Democratic Republic of Congo | GQ355474 | - | Analysis1 (835 bp) |
| *Plasmodium falciparum* | Humans |  | AY282930 | - | Analysis1 (835 bp) |
| *Hepatocystis sp* | Bats |  | DQ396527 | - | Analysis1 (835 bp) |
| *Hepatocystis sp* | Bats | Liberia | KF159712 | - | Analysis1 (835 bp) |
| *Hepatocystis sp* | Bats | Guinea | KF159683 | - | Analysis1 (835 bp) |
| *Hepatocystis sp* | Bats | Guinea | KF159695 | - | Analysis1 (835 bp) |
| *Hepatocystis sp* | African Monkeys | Ethiopia | AF069626 | - | Analysis1 (835 bp) |
| *Nycteria sp* | Bats | Guinea | KF159690 | - | Analysis1 (835 bp) |
| *Nycteria sp* | Bats | Ivory Coast | KF159720 | - | Analysis1 (835 bp) |
| *Haemoproteus columbae* | Birds |  | NC012448 | - | Analysis1 (835 bp) |
| *Parahaemoproteus vireonis* | Birds |  | FJ168561 | - | Analysis1 (835 bp) |
| *Polychromophilus sp* | Bats | Guinea | KF159714 | - | Analysis1 (835 bp) |
| *Polychromophilus sp* | Bats | Guinea | KF159700 | - | Analysis1 (835 bp) |
| *Polychromophilus melanipherus* | *Miniopterus schreibersii* | Switzerland | JN990708.1 | haplotype 3 | Analysis 2 (314 bp) |
| *Polychromophilus melanipherus* | *Miniopterus schreibersii* | Switzerland | JN990709.1 | haplotype 4 | Analysis 2 (314 bp) |
| *Polychromophilus melanipherus* | *Miniopterus schreibersii* | Switzerland | JN990710.1 | haplotype 5 | Analysis 2 (314 bp) |
| *Polychromophilus melanipherus* | *Miniopterus schreibersii* | Switzerland | JN990711.1 | haplotype 6 | Analysis 2 (314 bp) |
| *Polychromophilus melanipherus* | *Miniopterus inflatus* | Gabon | JQ995284.1 | haplotype 1 | Analysis 2 (314 bp) |
| *Polychromophilus melanipherus* | *Miniopterus inflatus* | Gabon | JQ995288.1 | haplotype 5 | Analysis 2 (314 bp) |
| *Polychromophilus melanipherus* | *Miniopterus inflatus* | Gabon | JQ995287.1 | haplotype 4 | Analysis 2 (314 bp) |
| *Polychromophilus melanipherus* | *Miniopterus inflatus* | Gabon | JQ995285.1 | haplotype 2 | Analysis 2 (314 bp) |
| *Polychromophilus melanipherus* | *Miniopterus inflatus* | Gabon | JQ995286.1 | haplotype 3 | Analysis 2 (314 bp) |
| *Polychromophilus melanipherus* | *Miniopterus manavi* | Madagascar | AY762071.1 | - | Analysis 2 (314 bp) |
| *Polychromophilus melanipherus* | *Miniopterus manavi* | Madagascar | AY762070.1 | - | Analysis 2 (314 bp) |
| *Polychromophilus melanipherus* | *Miniopterus manavi* | Madagascar | AY762074.1 | - | Analysis 2 (314 bp) |
| *Polychromophilus melanipherus* | *Miniopterus villiersi* | Guinea | KF159681.1 | - | Analysis 2 (314 bp) |
| *Polychromophilus melanipherus* | *Miniopterus villiersi* | Guinea | KF159699.1 | - | Analysis 2 (314 bp) |
| *Polychromophilus melanipherus* | *Miniopterus villiersi* | Guinea | KF159675.1 | - | Analysis 2 (314 bp) |
| *Polychromophilus murinus* | *Myotis goudoti* | Madagascar | AY762075.1 | - | Analysis 2 (314 bp) |
| *Polychromophilus murinus* | *Myotis daubentonii* | Switzerland | JN990712.1 | - | Analysis 2 (314 bp) |
| *Polychromophilus murinus* | *Myotis daubentonii* | Switzerland | HM055583.1 | - | Analysis 2 (314 bp) |
| *Polychromophilus sp.* | *Kerivoula hardwickii* | Cambodia | EF179354.1 | - | Analysis 2 (314 bp) |
| *Polychromophilus sp.* | *Pipistrellus aff. grandidieri* | Guinea | KF159714.1 | - | Analysis 2 (314 bp) |
| *Polychromophilus sp.* | *Neoromicia capensis* | Guinea | KF159700.1 | - | Analysis 2 (314 bp) |
| *Nycteria sp.* | *Rhinolophus landeri* | Guinea | KF159690 | - | Analysis 2 (314 bp) |
| *Nycteria sp.* | *Rhinolophus alcyone* | Guinea | KF159720 | - | Analysis 2 (314 bp) |
| *Polychromophilus melanipherus* | *Nycteribia schmidlii scotti, Penicilidia fulvida, Eucampsipoda africana, Brachytarsina allaudi* | Gabon | KU182361 | haplotype 1 | Analysis 1&2 |
| *Polychromophilus melanipherus* | *Nycteribia schmidlii scotti, Penicilidia fulvida, Raymondia huberi group* | Gabon | KU182362 | haplotype 2 | Analysis 1&2 |
| *Polychromophilus melanipherus* | *Nycteribia schmidlii scotti* | Gabon | KU182363 | haplotype 3 | Analysis 1&2 |
| *Polychromophilus melanipherus* | *Nycteribia schmidlii scotti* | Gabon | KU182364 | haplotype 4 | Analysis 1&2 |
| *Polychromophilus melanipherus* | *Penicilidia fulvida* | Gabon | KU182365 | haplotype 5 | Analysis 1&2 |
| *Polychromophilus melanipherus* | *Nycteribia schmidlii scotti* | Gabon | KU182366 | haplotype 6 | Analysis 1&2 |
| *Polychromophilus melanipherus* | *Nycteribia schmidlii scotti* | Gabon | KU182367 | haplotype 7 | Analysis 1&2 |
| *Polychromophilus melanipherus* | *Nycteribia fulvida* | Gabon | KU182368 | haplotype 8 | Analysis 1&2 |

Haplotypes are presented with the name used in the original studies. The last eight haplotypes (in bold) are from the present study and were submitted as a unique sequence to GenBank (with a single accession number), but some of them were detected in different bat fly species. Dash indicates non available information.

**Table S2:** Bat density per species and per cave, measured as the mean number of bats per night (5pm - 7am).

|  |  | **Cave** | | | |  | **ANOVA** | | |  |
| --- | --- | --- | --- | --- | --- | --- | --- | --- | --- | --- |
|  |  | Faucon | Zadie | Kessipoughou | Djibilong |  | ***F*** | ***df*** | ***P*** |  |
|  | *Coleura afra* | 10.2 ± 5.5 | Na | Na | Na |  | Na | Na | Na |  |
|  | *Hipposideros caffer* | 10.2 ± 2.2 | 16.7 ± 3.6 | 6.2 ± 1.5 | 5.5 ± 1.3 |  | 4.7 | 3 | 0.007 |  |
|  | *Hipposideros gigas* | 7.7 ± 3.8 | 15.4 ± 4.6 | 10.8 ± 2.2 | Na |  | 0.7 | 2 | 0.4 |  |
|  | *Miniopterus inflatus* | 17.2 ± 6.5 | Na | 5.9 ± 1.2 | 7.1 ± 1.2 |  | 5.1 | 2 | 0.009 |  |
|  | *Rousettus aegyptiacus* | Na | 22.4 ± 4.6 | 2.6 ± 0.6 | 1.0 ± 0.0 |  | 32.5 | 2 | < 0.001 |  |
|  | Total | 45.5 ± 1.5.1 | 51.0 ± 2.7 | 16.0 ± 2.1 | 10.9 ± 1.9 |  | 27.4 | 3 | < 0.001 |  |

**Na:** not applicable (i.e., bat species not recovered or free of bat fly infestation). **ANOVA**: Analysis of variance. ***F***: *F*-statistical value of the ANOVA. ***df***: degrees of freedom. ***P***: *P* value.

The total and species-specific density of captured bats varied significantly between caves, but for *H. gigas*.

**Table S3:** Seasonal variation of the number of bats collected in the Kessipoughou and Djibilong caves.

|  | **Months** | | | | | | | | | | | | ***χ^2^*** | ***df*** | ***P*** |
| --- | --- | --- | --- | --- | --- | --- | --- | --- | --- | --- | --- | --- | --- | --- | --- |
|  | 2012-05 | 2012-06 | 2012-07 | 2012-08 | 2012-09 | 2012-10 | 2012-11 | 2012-12 | 2013-01 | 2013-02 | 2013-03 | 2013-04 |  |  |  |
| **Kessipoughou** | 49 | * | 44 | 44 | 69 | 22 | 4 | 75 | 34 | 55 | 26 | 43 | 152.9 | 11 | < 0.001 |
| **Djibilong** | 10 | 83 | 19 | 33 | 30 | 10 | * | 1 | 26 | 27 | 6 | 6 | 267.3 | 11 | < 0.001 |

***χ^2^***: Chi-square test value. ***df***: degree of freedom. ***P***: *P* value. Star indicates month without sampling.

**Table S4:** Evaluation of the Poisson generalized linear mixed models fitted to estimate the effect of the bat-bat fly association.

|  |  | **Model terms (fixed effects)** | | |  |  |
| --- | --- | --- | --- | --- | --- | --- |
| **Model rank** | **Intercept** | **Bat species** | **Bat fly species** | **Bat x bat fly** | **AIC_C_** | **ΔAIC_C_** |
| 8 | -3,491 | **+** | **+** | **+** | 714.6 | -- |
| 4 | -2,491 | **+** | **+** |  | 1393.8 | 679.18 |
| 3 | -0,910 |  | **+** |  | 1394.7 | 680.04 |
| 2 | -1,741 | **+** |  |  | 1675.5 | 960.85 |
| 1 | -0,160 |  |  |  | 1676.5 | 961.87 |

Fixed effects included in the full model (Model No. 8) are the factors ‘Bat species’, ‘Bat fly species’ and their interaction. Crosses (+) indicate the occurrence of a term in the model. Random effects of all models are ‘Sites’ (caves) and ‘Month of Collection’, nested within ‘Bat species’. The models are ranked according to the increasing values of the second-order bias correction Akaike information criterion (AICc). The ΔAICc was calculated by comparing the AICc model with the model with the minimum AICc. The best model was selected when ΔAICc > 2 [44].

**Table S5:** Bat infestation rate (%) variations throughout the year.

|  | **May.2012** | **Jun.2012** | **jul.2012** | **Aug.2012** | **Sep.2012** | **Oct.2012** | **Nov.2012** | **Dec.2012** | **Jan.2013** | **Feb.2013** | **Mar.2013** | **Apr.2013** | ***χ^2^*** | ***df*** | ***P*** |
| --- | --- | --- | --- | --- | --- | --- | --- | --- | --- | --- | --- | --- | --- | --- | --- |
| **Both caves** |  |  |  |  |  |  |  |  |  |  |  |  |  |  |  |
| **M.i** | 66.7 | 44.2 | 60.8 | 60.5 | 70.0 | 76.9 | Na | 100 | 55.6 | 70 | 86.7 | 68.4 | 32.9 | 10 | **< 0.001** |
| **H.c** | 6.9 | 8.1 | 60 | 0.5 | 32.1 | 100 | Na | Na | 23 | 0 | 33.3 | 66.7 | 299.4 | 9 | **< 0.001** |
| **H.g** | 6.2 | Na | 11.7 | 100 | 33.3 | 41.2 | Na | 10.4 | 0 | 16.2 | 87.5 | 60 | 304.9 | 9 | **< 0.001** |
| **R.a** | 100 | 0 | 100 | 100 | 100 | Na | 22.2 | 75 | 75 | 100 | 100 | 0 | 249.7 | 10 | **< 0.001** |
| **Kessipoughou** |  |  |  |  |  |  |  |  |  |  |  |  |  |  |  |
| **M.i** | 66.7 | * | 66.7 | 62.5 | 70.7 | 75 | Na | 100 | 50 | 57.1 | 80 | 68.7 | 23.9 | 9 | **0.004** |
| **H.c** | 10.5 | * | 73.3 | 72 | 50 | Na | Na |  | 18.18 | 0 | 33.3 | Na | 140.1 | 6 | **< 0.001** |
| **H.g** | 6.2 | * | 11.7 | 100 | 33.3 | 58.3 | Na | 10.4 | 0 | 16.2 | 87.5 | 60 | 302.2 | 9 | **< 0.001** |
| **R.a** | 100 | * | 100 | 100 | 100 | Na | 22.3 | 75 | 100 | 100 | 100 | 0 | 157.4 | 9 | **< 0.001** |
| **Djibilong** |  |  |  |  |  |  |  |  |  |  |  |  |  |  |  |
| **M.i** | Na | 83.6 | 57.14 | 73.68 | 84.61 | 77.8 | * | 100 | 58.3 | 76.9 | 100 | 66.7 | 25.08 | 9 | **0.002** |
| **H.c** | 0 | 8.1 | 20 | 7.69 | 18.75 | 100 | * | Na | 50 | 0 | Na | 66.7 | 320.2 | 8 | **< 0.001** |
| **H.g** | Na | Na | Na | Na | Na | Na | * | Na | Na | Na | Na | Na | Na | Na | Na |
| **R.a** | Na | 0 | Na | Na | 100 | Na | * | Na | 50 | Na | 100 | Na | 20 | 2 | **< 0.001** |

**M.i**: *Miniopterus inflatus*; **H.c**: *Hipposideros caffer* complex; **H.g**: *Hipposideros gigas*; **R.a**: *Rousettus aegyptiacus*; **C.a**: *Coleura afra*. **df**: degrees of freedom. **Na**: not applicable (i.e., bat species not recovered or free of bat fly infestation).

***χ^2^***: Chi-square test value. ***df***: degree of freedom. ***P***: *P* value. Star indicates month without sampling. The mean infestation rates of bats varied significantly according to the bat species for the two caves taken together and also in each single cave.

**Table S6:** Mean number of bat flies per infested bat throughout the year.

|  | **May.2012** | **Jun.2012** | **Jul.2012** | **Aug.2012** | **Sep.2012** | **Oct.2012** | **Nov.2012** | **Dec.2012** | **Jan.2013** | **Feb.2013** | **Mar.2013** | **Apr.2013** | **ANOVA** | | |
| --- | --- | --- | --- | --- | --- | --- | --- | --- | --- | --- | --- | --- | --- | --- | --- |
|  |  |  |  |  |  |  |  |  |  |  |  |  | ***F*** | ***df*** | ***P*** |
| **Both caves** |  |  |  |  |  |  |  |  |  |  |  |  |  |  |  |
| **M.i** | 1.0 ± 0.0 | 2.7 ± 0.2 | 2.9 ± 0.5 | 2.1 ± 0.2 | 2.6 ± 0.2 | 3.8 ± 0.5 | Na | 1.0 ± 0.0 | 2.1 ± 0.3 | 3.1 ± 0.4 | 2.3 ± 0.4 | 2.7 ± 0.4 | 1.5 | 10 | 0.1 |
| **H.c** | 2.5 ± 1.5 | 1.0 ± 0.0 | 2.8 ± 0.8 | 2.5 ± 0.2 | 1.2 ± 0.2 | 1.0 ± 0.0 | Na | Na | 1.6 ± 0.6 | Na | 1.0 ± 0.0 | 3.0 ± 1.0 | 1.4 | 8 | 0.1 |
| **H.g** | 2.0 ± 0.0 | Na | 1.0 ± 0.0 | 2.0 ± 0.0 | 1.7 ± 0.4 | 1.2 ± 0.1 | Na | 1.0 ± 0.0 | Na | 1.3 ± 0.2 | 1.5 ± 0.2 | 1.3 ± 0.1 | 1.2 | 8 | 0.3 |
| **R.a** | 4.4 ± 0.5 | Na | 7.0 ± 0.0 | 2.0 ± 0.0 | 4.0 ± 1.5 | Na | 2.5 ± 1.5 | 3.1 ± 1.0 | 2.3 ± 0.8 | 2.0 ± 1.0 | 3.0 ± 0.7 | Na | 1.1 | 8 | 0.3 |
| **Kessipoughou** |  |  |  |  |  |  |  |  |  |  |  |  |  |  |  |
| **M.i** | 1.0 ± 0.0 | * | 2.8 ± 1.0 | 1.9 ± 0.4 | 2.4 ± 0.2 | 4.0 ± 0.0 | Na | 1.0 ± 0.0 |  | 3.6 ± 1.0 | 1.8 ± 0.2 | 2.7 ± 0.4 | 1.5 | 9 | 0.1 |
| **H.c** | 2.5 ± 1.5 | * | 2.9 ± 0.7 | 2.6 ± 0.2 | 1.3 ± 0.3 | Na | Na | Na | 2.0 ± 1.0 | Na | 1.0 ± 0.0 | Na | 1.1 | 5 | 0.3 |
| **H.g** | 2.0 ± 0.0 | * | 1.0 ± 0.0 | 2.0 ± 0.0 | 1.7 ± 0.4 | 1.2 ± 0.1 | Na | 1.0 ± 0.0 | Na | 1.3 ± 0.2 | 1.5 ± 0.2 | 1.3 ± 0.1 | 1.2 | 8 | 0.3 |
| **R.a** | 4.4 ± 0.5 | * | 7.0 ± 0.0 | 2.0 ± 0.0 | 4.5 ± 2.5 | Na | 2.5 ± 1.5 | 3.1 ± 1.0 | Na | 2.0 ± 1.0 | 3.3 ± 0.8 | Na | 1.1 | 8 | 0.3 |
| **Djibilong** |  |  |  |  |  |  |  |  |  |  |  |  |  |  |  |
| **M.i** | Na | 2.7 ± 0.2 | 2.8 ± 1.0 | 2.2 ± 0.3 | 3.1 ± 0.6 | 3.7 ± 0.8 | * | 1.0 ± 0.0 | 2.3 ± 0.4 | 2.9 ± 0.4 | 3.2 ± 1.1 | 3.0 ± 2.0 | 0.6 | 9 | 0.7 |
| **H.c** | Na | 1.0 ± 0.0 | 2.0 ± 0.0 | 1.0 ± 0.0 | 1.0 ± 0.0 | 1.0 ± 0.0 | * | Na | 1.0 ± 0.0 | Na | Na | 3.1 ± 1.0 | 2.8 | 6 | 0.1 |
| **H.g** | Na | Na | Na | Na | Na | Na | * | Na | Na | Na | Na | Na | Na | Na | Na |
| **R.a** | Na | Na | Na | Na | 3.0 ± 0.0 | Na | * | Na | 4.0 ± 0.0 | Na | 2.0 ± 0.0 | Na | Na | Na | Na |

**M.i**: *Miniopterus inflatus*; **H.c**: *Hipposideros caffer* complex; **H.g**: *Hipposideros gigas*; **R.a**: *Rousettus aegyptiacus*; **C.a**: *Coleura afra*. **ANOVA**: Analysis of variance. ***F***: *F*-statistical value of the ANOVA. ***df***: degrees of freedom. ***P***: *P* value. **Na**: not applicable (i.e., bat species not recovered or free of bat fly infestation). Star indicates month without sampling.

The result of the ANOVA shows that the mean number of bat flies per infested bat did not vary significantly (***P*** > 0.5) during the year in the two caves taken together and also in each cave.

**Table S7:** Number of Infected Pools (NIP) and Maximum Likelihood Estimates of Infection Rate (MLE-IR) of bat fly species.

|  | NIP | MLE-IR (%) |
| --- | --- | --- |
| *N. schmidlii scotti* | 64 | 12 |
| *P. fulvida* | 8 | 11.3 |
| *E. africana* | 1 | 0.5 |
| *R. huberi group* | 1 | 1.8 |
| *B. allaudi* | 1 | 1.7 |

**Table S8:** Haplotype of the *P. melanipherus* mtDNA cytochrome b sequences found in *Nycteribia schmidlii scotti* and *Penicilidia fulvida* bat flies collected in the four caves in Gabon.

| **Haplotypes** |  | ***cyt b* polymorphic sites** | | | | | | | | | | | | | | | | | | | | |  | **Haplotype frequencies** | | | |
| --- | --- | --- | --- | --- | --- | --- | --- | --- | --- | --- | --- | --- | --- | --- | --- | --- | --- | --- | --- | --- | --- | --- | --- | --- | --- | --- | --- |
|  |  | 27 | 36 | 105 | 115 | 129 | 193 | 225 | 256 | 257 | 280 | 339 | 345 | 371 | 390 | 418 | 450 | 456 | 595 | 729 | 784 | 799 |  | n | Faucon | Kessipoughou | Djibilong |
| KF159699.1 |  | A | T | T | T | G | C | T | T | T | G | A | T | G | C | T | T | T | A | A | C | C |  |  |  |  |  |
| Hap_1 [KU182361] |  | A | T | A | T | G | C | T | C | T | G | A | T | G | C | T | T | C | A | A | T | T |  | 35 | 2 | 15 | 18 |
| Hap_2 [KU182362] |  | . | A | A | C | . | . | . | T | . | . | . | C | . | T | . | . | . | G | T | . | C |  | 26 | 3 | 7 | 16 |
| Hap_3 [KU182363] |  | T | . | A | . | . | . | C | . | . | . | . | . | . | . | C | C | . | G | . | . | C |  | 9 | 0 | 3 | 6 |
| Hap_4 [KU182364] |  | . | A | A | C | . | . | . | T | . | . | . | C | A | T | . | . | . | G | T | . | C |  | 1 | 0 | 0 | 1 |
| Hap_5 [KU182365] |  | . | . | A | . | . | T | . | T | . | A | T | . | . | . | . | . | T | G | . | C | C |  | 1 | 0 | 1 | 0 |
| Hap_6 [KU182366] |  | T | . | A | . | . | . | C | . | G | . | . | . | . | . | C | C | . | G | . | . | C |  | 1 | 0 | 0 | 1 |
| Hap_7 [KU182367] |  | . | . | A | . | A | . | . | . | . | . | . | . | . | . | . | . | . | . | . | . | . |  | 1 | 0 | 1 | 0 |
| Hap_8 [KU182368] |  | T | . | A | . | . | . | C | . | . | . | . | . | . | . | C | . | . | G | . | . | C |  | 1 | 0 | 0 | 1 |

Only polymorphic positions are shown, and are numbered based on the published *P. melanipherus* cyt b sequence [KF159699.1] from *Miniopterus villiersi* collected in Guinea [1]. In brackets: GenBank accession numbers.

**Reference**

1. Schaer J, Perkins SL, Decher J, Leendertz FH, Fahr J, Weber N, et al. High diversity of West African bat malaria parasites and a tight link with rodent Plasmodium taxa. Proc Natl Acad Sci U S A. 2013;110(43):17415–9.

**Legend for supplemental figure**

**Figure S1.** *Polychromophilus melanipherus* haplotype distribution.

Pie charts showing the *P. melanipherus* haplotype distribution in the Faucon, Kessipoughou and Djibilong caves. No *P. melanipherus* infection was detected in bat flies collected from bats captured in Zadie cave.
