# Supplementary figures and images for: Bat flies (Diptera: Nycteribiidae and Streblidae) infesting cave-dwelling bats in Gabon: diversity, dynamics and potential role in Polychromophilus melanipherus transmission
Source: Parasit Vectors. 2016 Jun 10;9:333. doi: 10.1186/s13071-016-1625-z (PMC4902993; doi:10.1186/s13071-016-1625-z)

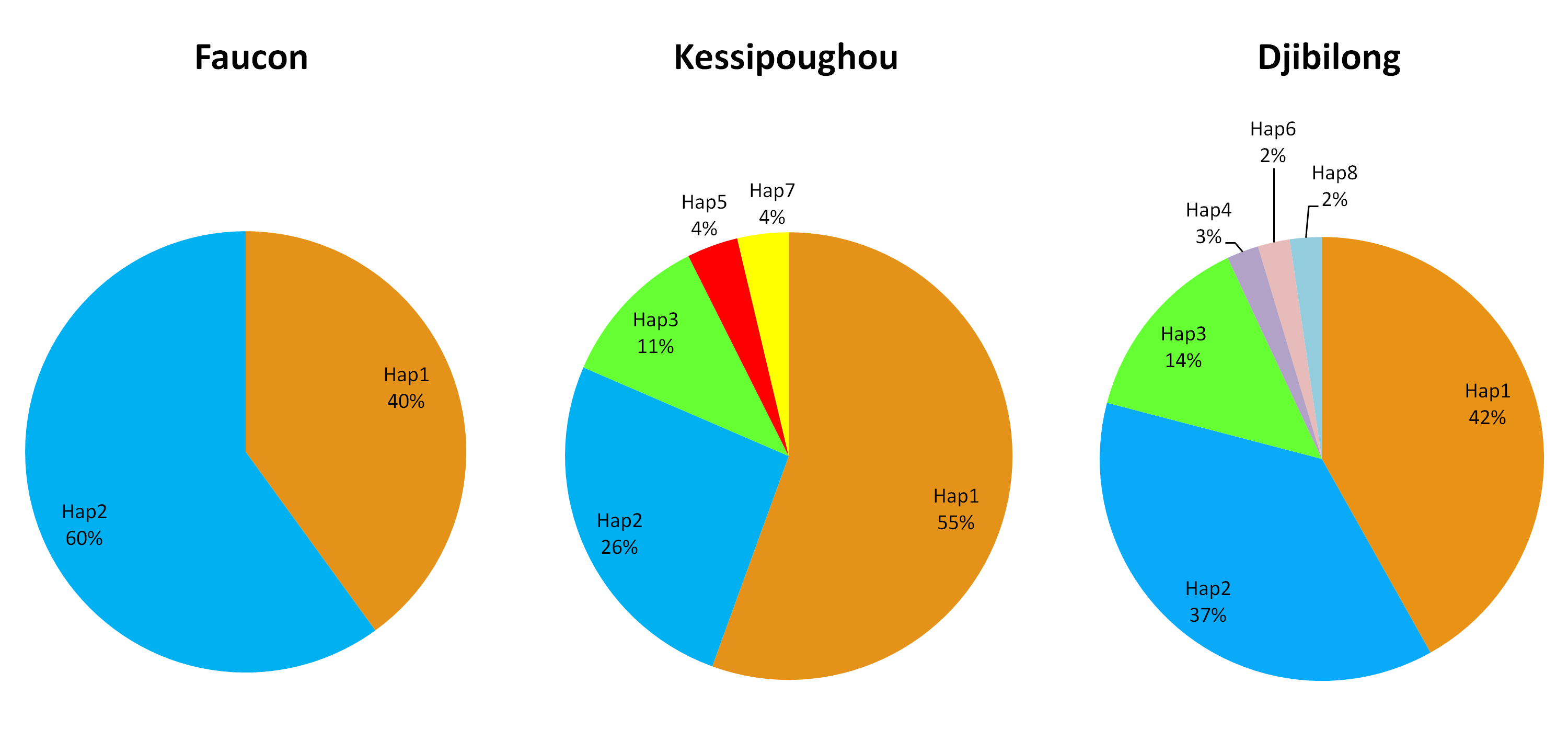

Supplement: Additional file 2: Figure S1. — Polychromophilus melanipherus haplotype distribution. Pie charts showing the P. melanipherus haplotype distribution in the Faucon, Kessipoughou and Djibilong caves. No P. melanipherus infection was detected in bat flies collected from bats captured in Zadie cave. (TIF 845 kb) [file 13071_2016_1625_MOESM2_ESM.tif]
